# Can Non-Beak Treated Hens be Kept in Commercial Furnished Cages? Exploring the Effects of Strain and Extra Environmental Enrichment on Behaviour, Feather Cover, and Mortality

**DOI:** 10.3390/ani6030017

**Published:** 2016-02-25

**Authors:** Krysta L. H. Morrissey, Sarah Brocklehurst, Laurence Baker, Tina M. Widowski, Victoria Sandilands

**Affiliations:** 1 Monogastric Science Research Centre, Scotland’s Rural College (SRUC), Auchincruive Estate KA6 5HN, UK; laurence.baker@sruc.ac.uk (L.B.); vicky.sandilands@sruc.ac.uk (V.S.); 2 Department of Animal Biosciences, University of Guelph, Guelph ON N1G 2W1, Canada; twidowsk@uoguelph.ca; 3 Campbell Centre for the Study of Animal Welfare, University of Guelph, Guelph ON N1G 2W1, Canada; 4 Biomathematics and Statistics Scotland, James Clerk Maxwell Building, King’s Buildings, Peter Guthrie Tait Road, Edinburgh EH9 3FD, UK; sarah.brocklehurst@bioss.ac.uk

**Keywords:** beak trimming, beak treatment, laying hen, injurious pecking, furnished cage, feather cover, environmental enrichment

## Abstract

Commercial laying hens are prone to injurious pecking (IP), a common multifactorial problem. A 2 × 2 × 2 factorial design assessed the effects of breed (Lohmann Brown Classic (L) or Hyline Brown (H)), beak treatment (infra-red treated (T) or not (NT)), and environment (extra enrichment (EE) or no extra enrichment (NE)) on mortality, behaviour, feather cover, and beak shape. Hens were allocated to treatments at 16 weeks of age and data were collected every four weeks from age 19 to 71 weeks. Data were analysed in Genstat using mixed models. L hens had higher all and IP-related mortality than H hens (*p* < 0.003), whilst NT hens had higher mortality than T hens but only due to culling of whole cages (*p* < 0.001). Feather cover for L hens deteriorated more quickly with age at most body sites than H hens (age × breed × body site *p* < 0.001). For NT hens, feather cover was worse at most body sites (beak treatment × body site *p* < 0.001), and worsened more quickly with age (age × beak treatment *p* = 0.014) than T hens. L and NE hens performed more bird-to-bird pecking than H and EE hens, respectively (breed *p* = 0.015, enrichment *p* = 0.032). More damage to mats and ropes was caused by L and NT hens than by H and T hens, respectively (age × breed *p* < 0.005, beak treatment *p* < 0.001). Though H hens had fewer mortalities and better feather cover, breed effects may have been influenced by farm management practices, as they may have been better suited to H than L hens. Though EE hens performed less bird-to-bird pecking, the enrichments were less effective at reducing feather cover damage and mortality than expected.

## 1. Introduction

Injurious pecking (IP) is currently the most problematic behavioural issue facing the poultry industry as it impacts on bird welfare as well as production economics [1,2]. IP is categorised as bird-to-bird pecking that results in plumage damage, feather loss, and tissue damage, and includes gentle and severe feather pecking as well as cannibalism [3]. Aggressive pecking is not generally considered within the IP umbrella as it has a different etiological basis [4,5], even though high levels of aggression can also cause tissue damage and reduce welfare. In general, the prevalence of IP in a flock is thought to reflect unfulfilled behavioural needs, so it is not just a welfare problem for the victims [5]. Although IP is not a new problem [6,7], it is still notoriously difficult to prevent and treat.

IP is a multifactorial problem, with a wide range of risk factors, including genetics and environment [4]. Genetic differences in the propensity to perform IP is evident between breeds [7,8] as well as between two divergently selected lines of a single strain (*i.e.*, high and low-feather peckers) [9]. The heritability of IP behaviour ranges from 0.20 to 0.65 [9,10], meaning that selection against this behaviour could be introduced into commercial breeding programmes to reduce IP. However, this type of commercial selection may only prove beneficial as a long-term goal as balances between behavioural traits and egg production are perfected. There has been some indication that selection against IP increases egg production, but reduces egg quality [11]. Modern selection methods are beginning to incorporate group performance into breeding programs which could include some way to identify groups of high feather peckers [12,13]. In theory, group selection incorporates behavioural and physiological traits that would support high levels of productivity [14]. In addition, identifying differences between breeds or strains that are currently available will help producers chose the most suitable breed for the housing system in use.

Providing birds with the opportunity to forage (e.g., by providing straw, whole grain in litter, *etc.*) reduces the risk of feather pecking [6,15,16]. On commercial free range and barn systems, maintaining good litter quality can reduce the risk of gentle feather pecking [17]. However, for caged systems, providing suitable foraging substrates can be challenging even though it might be of more importance given the relatively barren environment of a cage. Various types of plastic toys can reduce aggressive pecks [18,19] and providing string enrichments to hens can reduce feather pecking, thereby improving feather cover [20]. In particular, white string enrichments have been shown to be attractive to chicks [21] and hens pecked more frequently at white or yellow string than chains and beads or blue or orange string [22,23].

Currently, using some method of beak treatment remains the most commonly used and most effective and reliable method of reducing the damage caused by IP [7,24,25]. This is not to say that pecking is not an issue in beak treated flocks, but that the damage they are able to cause is lessened. There are two main methods of treating beaks for poultry: hot blade trimming and infra-red treatment. Infra-red beak treatment has fewer adverse effects on chicks than hot blade trimming with regards to neuroma formation and symptoms of chronic pain [26,27,28]. Despite these benefits, infra-red beak treatment is still deemed a mutilation and some European countries have altogether banned any type of beak treatment [29]. Other governmental agencies, like the Department for Environment, Food and Rural Affairs (DEFRA) in England, aimed to ban the practice but then withdrew due to advisors indicating that subsequent damage caused by pecking in intact-beak birds would be too great a risk to welfare [30]. Although infrared beak treatment will continue in England and the rest of the United Kingdom (UK) for the foreseeable future, the issue may rise again, whereby the practice could stop voluntarily or be prevented through legislation.

Though there is a wealth of information pertaining to the aetiology, risk factors, and prevention of IP, there is not a great deal of information pertaining to performance in furnished cage systems. The ban on conventional cages within the European Union in 2012 with Council Directive 1999/74/EC [31] unearthed the need for more housing management information specific to this type of housing. Furnished cages were initially designed to house relatively small groups of hens (e.g., 20), however the trend now is for larger group sizes, which may have implications for social behaviour and development of IP. Therefore, understanding how to manage non-beak treated hens in these systems is important, especially if legal or voluntary bans on beak treatment take effect across the EU. Therefore, the goal of this study was to measure the effect of breed and extra environmental enrichment on behaviour and welfare between beak treated and non-beak treated hens.

## 2. Materials and Methods

### 2.1. Ethical Considerations

This study was conducted under a Home Office licence according to the Animals (Scientific Procedures) Act 1986 and was approved by Scotland’s Rural College’s animal ethics committee (AU AE 35-2012). Due to the high risk nature of housing non-beak treated hens, an overall cage threshold for intervention was defined as: “two or more birds from one cage die or are culled due to pecking related damage”. Appropriate intervention included hot blade beak trimming or culling the remaining hens within the affected cage.

### 2.2. Animals

A total of 5120 laying hens of either Hyline Brown (2560) or Lohmann Brown Classic (2560) were used. Half of the chicks of each strain were beak treated at day-old at the hatchery using an infrared technique (Nova-Tech Engineering, Willmar, MN, USA). From 0 to 7 weeks of age, pullets were reared in groups of 1280 in deep-littered floor pens at a commercial farm in the UK, according to breed and beak treatment. From 7 to 16 weeks of age, the pullets were housed in small colony cages designed for pullets. Pullets had *ad libitum* access to commercial rearing diets and water and were reared following standard commercial schedules for temperature, lighting, and vaccinations.

### 2.3. General Husbandry

For the duration of this study (16 to 71 weeks of age), all hens were housed in 80-bird furnished cages (4.81 m × 1.26 m or 758 cm^2^/bird, Tecno Poultry Equipment S.p.A., Marsango di Campo San Martino, Italy) at a commercial farm in the UK. The shed used in this experiment contained seven banks of cages, each ten cages high and 22 cages long (a total of 1540 cages). The shed was split into two levels, with a suspended floor separating the fifth and sixth tiers. Lights were suspended from the ceiling and bobbed between three vertical positions every 20 min. The bottom tiers on each level were excluded from the experiment as they were not illuminated as brightly as the others (tier 1 and 6, mean: 1.5 lux *vs.* remaining tiers mean: 8.8 lux). To mimic dawn and dusk, lights at both ends of the shed were switched on 30 min prior to lights-on and 30 min after lights-off. Sixty-four cages (eight cages long × eight tiers) were used in this experiment and they were all located in the exact centre of the shed. Each cage contained the furnishings required by law (European Council Directive 1999/74/EC), including two nesting areas (each measuring 60 cm × 60 cm), perches (15 cm/bird, at two different heights) and two scratching areas (each measuring 38 cm × 24 cm). For both preventive and therapeutic reasons, hens were treated for red mite infestations throughout the lay period (Milben Ex, applied topically). All hens had *ad libitum* access to water and a standard commercial layer’s mash, delivered by automatic feed hoppers eight times per day.

### 2.4. Experimental Treatments

The experiment was designed as a 2 × 2 × 2 factorial, with breed (Hyline Brown (H, which was the breed used in the rest of the shed) or Lohmann Classic (L)), beak treatment (infra-red treated (T) or not treated (NT)), and extra enrichments (no extra enrichment (NE) or extra enrichment (EE)) as the three main factors. Upon arrival to the laying farm at 16 weeks of age, birds were allocated to the cages in groups of 80 hens, with eight cages (640 birds) per treatment. The treatments were systematically allocated to cages so that the eight treatments and the two levels of each of the three factors balanced spatially, with each column and each tier containing each treatment. Half of the cages had been fitted with extra environmental enrichments, including eight polypropylene ropes (8 mm diameter, 40 cm long), two pecking mats (each measuring 30 cm × 10 cm) and two beak blunting boards (each measuring 30 cm × 10 cm), evenly distributed throughout the cage. The pecking mats comprised of a combination of compressed wood chips and biodegradable glue on a plastic mesh backing (ROWA, Melle, Germany). The beak blunting boards were made up of an abrasive paste (S N Supplies, Lincoln, UK) previously used in beak blunting trials [32,33] which was painted onto a Perspex^®^ backing. Four bolts were drilled into each blunting board to act as a shiny attractant for the birds. The pecking mats and blunting boards were hung vertically and fixed to the fronts of the cages. The ends of each rope were lightly cauterized to slow destruction and prevent birds from swallowing rope fibres. The ropes were doubled over and secured to the cage top so that they hung from the cage ceiling to 20 cm below it.

### 2.5. Data Collection

Every 4 weeks, from 19 to 71 weeks of age, two observers visited the farm over two consecutive days to collect data pertaining to bird behaviour, feather cover and extra enrichment wear and tear, resulting in 14 farm visits in total. On the first day, scan sampling for injurious pecking behaviour, as well as assessment of feather cover and extra enrichment damage were collected. Both observers were present and each observed either the top four or bottom four tiers, alternating at consecutive visits. The second day was entirely devoted to live focal bird sampling for all types of oral behaviour (with only one observer present).

#### 2.5.1. Mortality

Birds were examined by stock workers on a daily basis and mortality (deaths or culls) was recorded as necessary. Hens were assessed for cause of death (or reason for cull) and whether it was related to IP. To prevent misclassification, from age 48 weeks, the carcasses were chilled and brought to a veterinarian for post-mortem examination, from which the cause of death was established.

#### 2.5.2. Behavioural Measures

One scan sample per cage was performed on the first day of each farm visit to record the number of hens performing all types of bird-to-bird pecks (Table 1). Only the hens in the west half of the cage (*i.e.*, hens between the front and centre line of the cage) were observed due to limited visibility. Within each tier, the cages were always observed in order from south to north. However, the order with which the tiers were observed was balanced using four Latin Squares on the basis of a maximum of 16 visits (and eight tiers), though the last two visits were dropped as the birds were depleted just after the fourteenth visit (approximately 73 weeks of age). Scan sample data were gathered from 9:00 a.m. to 11:00 a.m.

For focal behaviour sampling performed on the second day of each farm visit sampling cages in the same order as described above, a handheld machine (Psion Workabout Pro^3^, Motorola Solutions, Schaumburg, IL, USA) was used to collect data via Pocket Observer software (Observer XT v11, Noldus Information Technology, Wageningen, The Netherlands). One hen per cage was observed during each visit between 9:00 a.m. and 4:00 p.m. for a maximum of 5 min (which was shortened to 4 min from the second visit onwards to accommodate the farm’s working hours). All pecking related behaviours were recorded (Table 1). To start an observation, one focal hen was systematically chosen from each cage based on its proximity to one of four pre-determined locations. To reduce selection bias, these locations or their equivalent in NE cages (nearest south nest box, second rope, blunting board or pecking mat) were distributed as evenly as possible along the length of the visible side of the cage and starting locations and observation order were balanced as evenly as possible across visits. If the observer could not see the original focal hen for the entire observation, another hen was chosen from the same original starting location and the observation was continued. This change in bird was recorded. The bottom tiers on each level (tiers 2 and 7) were observed from the ground, whereas it was necessary to use a mobile trolley to view all of the other tiers.

#### 2.5.3. Feather Cover

Following behavioural scan sampling, feather cover was assessed for four hens per cage, travelling along the tiers from north to south, following the same pattern as the order for scan sampling, although cage order within tier was reversed. Observed hens were selected from pre-determined locations within the cage (nearest north perch, north side of scratch mat, south side of scratch mat, south perch). Hens were observed from outside the cage and given a feather cover score from 0 to 5 (where 0 was no damage; see [34]) for each visible body site (head, comb, neck, breast, back, both wings, rump, thigh, belly, and tail). The comb was given a score based on the number of scratches present up to a maximum of 5 (*i.e.*, a comb with more than five scratches would still be given a score of 5) and was included in the analyses even though damage here most likely reflects aggression and not feather pecking.

#### 2.5.4. Extra Enrichment Use

Damage to each extra enrichment was assessed and given a score at each visit. Ropes were given a score from 1 to 3 (1 = no evidence of use; 2 = mild use, <50% frayed; 3 = moderate to vigorous use, ≥50% frayed). Mats and boards were given a score from 1 to 5 (1 = no evidence of use; 2 = mild use, <25% worn; 3 = mild to moderate use, 25%–50% worn; 4 = moderate use, 51%–75% worn; 5 = vigorous use, >75% worn). An extra enrichment was replaced once its individual score reached a certain threshold of damage (score ≥ 4 for mats and boards, score = 3 for ropes).

#### 2.5.5. Beak Shape

**Figure 1:**
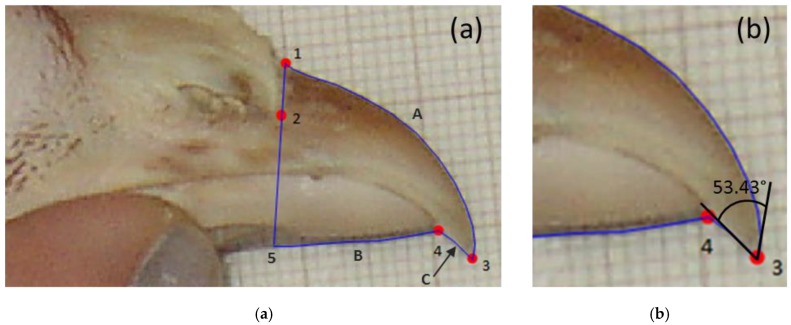
Screenshot of tpsDig2 software used to landmark reference points on the beak, and to perform precise measurements of upper (A) and lower mandible (B) lengths as well as overhang (C) length (**a**) and to measure the inner angle of the beak tip (**b**).

At 64 weeks of age, beaks of four hens per cage were photographed from the side on a background of graph paper. Using tpsDig2 software (SUNY Stony Brook Morphometrics, Stony Brook, NY, USA), four landmark reference points were added to each of the photos and the length of the upper and lower mandibles were traced (Figure 1a, method adapted from [35]). In addition, the amount of upper or lower mandible overhang (negative numbers if lower mandible extended beyond upper mandible) and beak tip angles were measured (Figure 1b). The single observer analysing beak photos was blind to treatment.

### 2.6. Statistical Analyses

Tests reported from linear mixed models (LMMs) and generalised mixed models (GLMMs) are approximate *F* tests when these are available but otherwise Wald tests are reported, with threshold for statistical significance α = 0.05. Age was fitted as a covariate in the fixed effects with linear, quadratic and cubic functions of age included, when appropriate. Higher level interactions were omitted from fixed effects in GLMMs when required due to sparseness in the response variable. Negligible random effects were also omitted from GLMMs to aid computation. Means (± standard error (SE), as well as standard error of differences (SEDs)) reported are estimated from the mixed models and when data are transformed, or for GLMMs, estimates from models of mean (mean + SE, mean − SE) are back transformed if applicable to aid interpretation. All data were analysed in Genstat (16th edition, VSN International, Hemel Hempstead, UK).

#### 2.6.1. Mortality

Two cages were removed from the study at 48 weeks of age due to pecking related mortality exceeding the permitted threshold of two hens per cage (or 2.5%) and all remaining hens in these cages were culled by cervical dislocation to avoid any further welfare problems. The total mortality data over the whole trial period (age 16 to 71 weeks) were analysed both excluding these healthy birds that were culled (“minimum” mortality) and including them (“maximum” mortality) and these were further divided into mortality due to all causes (*i.e.*, “all” mortality) and those that were deemed to be related to IP. The resulting four types of proportions of dead birds were analysed by fitting GLMMs to the counts of dead birds per cage out of binomial total 80, with binomially distributed errors and logit link function. Tier was included as a random effect in all models. Fixed effects were breed, beak treatment, and enrichment, including all interactions for all mortality and two way interactions for IP related mortality.

#### 2.6.2. Behaviour

Scan sampling data were too sparse for a statistical analysis; however, summary statistics are presented.

Bird-to-bird pecks during focal sampling were quite infrequent and therefore were analysed as a group as were pecks directed at extra enrichments (Table 1). Each type of peck (bird-to-bird, extra enrichment, other) as well as the number of bird changes were analysed by fitting LMMs to the rate (count per min) transformed as required (natural logarithm + 0.1 for bird-to-bird pecks and extra enrichment pecks, natural logarithm + 1 for number of bird changes, square root for “other” pecks). Age within cage within tier were included as random effects in all models. Fixed effects were starting location, age (as a covariate), breed, beak treatment, and enrichment (including up to three-way interactions between treatment factors and age). The model for extra enrichment pecks was applied to data from cages with extra enrichments only and excluded enrichment from the fixed effects.

#### 2.6.3. Feather Cover

The proportions of positive feather cover scores were analysed by fitting a GLMM to the binary variable of whether each score was greater than 0, or not, out of binomial total 1, with binomially distributed errors and logit link function. Tier and cage, age within tier and within cage, and bird location within cage and age (*i.e.*, the selected bird) were included as random effects. Fixed effects were observer, body site, age (as a covariate), breed, beak treatment, and enrichment. For the body site fixed effect, the two wing sites were combined into one level called “wing” and low scoring sites were also grouped together (head, comb, back, rump, breast and belly) and classified as “the rest”. Interactions of observer with body site, and up to three way interactions of body site with age, and each of the treatment factors were included. However, interactions with beak treatment were limited to two way as the data were sparse.

#### 2.6.4. Extra Enrichment Use

When any of the extra enrichments were replaced due to wear, its damage score was reset to “1”. Therefore, to account for changes in scores with age, the scores for each of the extra enrichments were cumulated over time. LMMs were fitted to the cumulative damage scores transformed (natural logarithm + 1) for ropes and mats, but not to scores for the beak blunting boards as they typically scored “1” at each visit and therefore the data were too sparse to support a statistical analysis. The rope score data is only available from 19 to 51 weeks of age because one rope dislodged from the cage at 51 weeks, giving rise to concern about damage to the egg belt mechanism, and were thus removed. Age within cage within tier, and location within cage (*i.e.*, each individual rope or mat) were included as random effects in all models. Fixed effects were observer together with age (as a factor), breed, beak treatment, and their interactions.

#### 2.6.5. Beak Shape

LMMs were fitted to beak measurements of lower and upper mandible lengths, beak tip angle (natural logarithm transformed), and overhang length (not transformed). Bird within cage within tier were included as random effects in all models. Fixed effects were breed, beak treatment, enrichment, and their interactions. For upper and lower mandible measurements, 19% of photos were not useable (*i.e.*, too blurry) and were thus excluded from analysis. For beak tip angle, a subset of the data was analysed that included only clear photos of NT hens (77%) as beaks of T hens were too blunt to measure an angle, and beak treatment was removed from the fixed effects. For the overhang length, a subset of the data (73%) was analysed that included only clear photos in which the hens had their beaks fully closed.

## 3. Results

### 3.1. Mortality

By 48 weeks of age, two cages (L-NT-NE and L-NT-EE) had surpassed the pecking related mortality threshold and required remedial action. The first cage (L-NT-NE) had a total of ten hens found dead (12.50%), seven (8.75%) of which had been identified by the stock worker as being related to IP (mostly vent pecking). The IP-related deaths occurred between 43 to 48 weeks of age. The second cage (L-NT-EE) had a total of eight hens found dead (10.00%), five (6.25%) of which had been identified by the stock worker as being related to IP. These IP-related deaths occurred between 28 to 44 weeks of age. All remaining hens from both cages were removed from the study and culled.

L hens had significantly higher proportions of both minimum and maximum mortality than H hens for both all (*p* < 0.002) and IP-related (*p* < 0.003) deaths (Table 2). There was a significant beak treatment effect for maximum mortality data only, as NT hens had higher proportions of mortality than the T hens for both all (*p* < 0.001) and IP-related (*p* < 0.001) mortality. There were no differences between NE and EE hens for minimum and maximum all or IP-related mortality. There were marginally significant breed × beak treatment interactions for minimum all (*p* = 0.039), maximum all (*p* = 0.013), and maximum IP (*p* = 0.025) mortality. H-T hens had fewer minimum all mortalities than H-NT hens, but no such pattern was observed for L hens, whilst for maximum all and IP mortality L-NT hens had substantially higher mortality than the other three groups. There was a marginally significant beak treatment × enrichment interaction for maximum all mortality (*p* = 0.037), with the increase in mortality in NT-EE compared to T-EE slightly larger than the increase in mortality in NT-NE compared to T-NE.

### 3.2. Behaviour

Out of a possible 35,360 occurrences during scan sampling, only 43 observations (0.12%) were made of hens performing some type of bird-to-bird pecking. Of these 43, 74.4% were gentle feather pecks, 14% were vigorous feather pecks, and 11.6% were aggressive pecks. The differences appeared to be minimal between treatments, but were too sparse to statistically analyse.

Similarly, relatively few occurrences of bird-to-bird pecking were observed during focal sampling, including no observations of cannibalistic or vent pecks (Table 3). Other pecks were the most commonly observed, and gentle feather pecks, pecks received by the focal hen, and rope pecks were the only other behaviours observed more than 1% of pecks (Table 3).

In general, bird-to-bird pecks increased with age (*p* < 0.001), and peaked around 47 weeks of age. Observations that began with hens near the nest and mat resulted in fewer bird-to-bird pecks than for hens beginning near the rope, with the board intermediate (starting location effect *p* < 0.001; Table 4). L hens performed more bird-to-bird pecking than H hens (*p* = 0.015) and NE hens performed more bird-to-bird pecking than EE hens (*p* = 0.033). No significant differences due to beak treatment were observed (*p* = 0.248).

For pecks directed at the extra enrichments, there was an effect of age (*p* < 0.001) and starting location as pecks directed at the extra enrichments generally decreased over time and were more often observed near the rope and mat than the nest and board (starting location effect *p* < 0.001). There were no statistically significant effects of other treatment factors (*p* > 0.207). For other pecks, there was an effect of age (*p* < 0.001) as they generally increased over time. NT appeared to have fewer other pecks for L hens but not for H hens (breed × beak treatment *p* = 0.036 (SED = 0.161): means estimated from LMM back transformed onto rate scale and estimated means ± SE on transformed scale: H-T 3.873 (1.968 ± 0.188), H-NT 4.256 (2.063 ± 0.188), L-T 5.258 (2.293 ± 0.188), L-NT 3.595 (1.896 ± 0.190) pecks per minute). Finally, the rate of bird changes per observation was higher for L hens than for H hens (*p* < 0.001; Table 4) and slightly higher for NT hens than for T hens (*p* = 0.048).

### 3.3. Feather Cover

**Figure 2:**
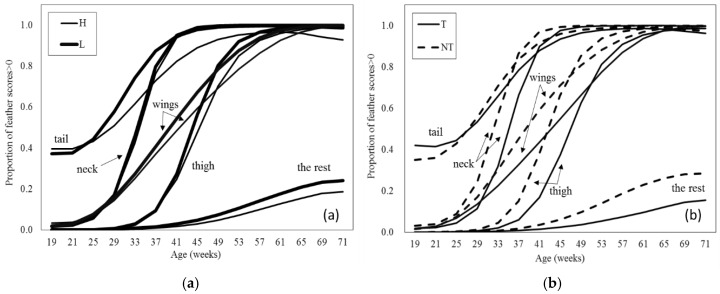
Mean proportion feather cover score >0 (back transformed from logit scale) estimated from generalised mixed models (GLMM) by (**a**) breed (H: Hyline Brown, L: Lohmann Brown Classic) and (**b**) beak treatment (T: Infra-red treated, NT: Not treated), and by body site and age.

As would be expected, feather damage increased with age (*p* = 0.001). Feather cover for L hens deteriorated more quickly than for H hens at all body sites except neck (breed *p* < 0.001, age × breed × body site *p* < 0.001; Figure 2a). For the other sites, tail scores appear to differ the most between the two breeds, with the rest having the smallest difference. The proportion of scores > 0 was analysed, so this reached the maximum, 1, for all body sites (except “the rest”) at about 60 weeks of age. Therefore, the differences in feather cover for most sites occurred in the middle portion of this experiment although for the rest the difference between the two breeds was still apparent at the end of the study. Feather cover in most sites worsened more quickly with age for NT hens than for T hens (beak treatment *p* < 0.001, age × beak treatment *p* = 0.014) and NT scored higher than T for each of the body sites except tail (beak treatment × body site *p* < 0.001) from 25 to 37 weeks up to approximately 60 weeks of age (Figure 2b). As with breed, apparent differences for most sites occurred in the middle portion of this experiment, although for the rest the difference between the two breeds was still apparent at the end of the study. Though there was no overall effect of enrichment (*p* = 0.831), EE hens had better wing feather quality, but poorer thigh feather quality, with no differences for neck, tail, or the rest (body site × enrichment *p* = 0.005) and extra enrichments did not affect feather scores for T hens, but appear to have had a negative impact on feather scores for NT hens (beak treatment × enrichment *p* = 0.033 (SED = 0.170); means estimated from GLMM back transformed on to proportion scale and estimated means ± SE on logit scale: T-NE 0.622 (0.499 ± 0.148), T-EE 0.608 (0.438 ± 0.154), NT-NE 0.774 (1.233 ± 0.151), and NT-EE 833 (1.604 ± 0.159)).

### 3.4. Extra Enrichment Use

**Figure 3:**
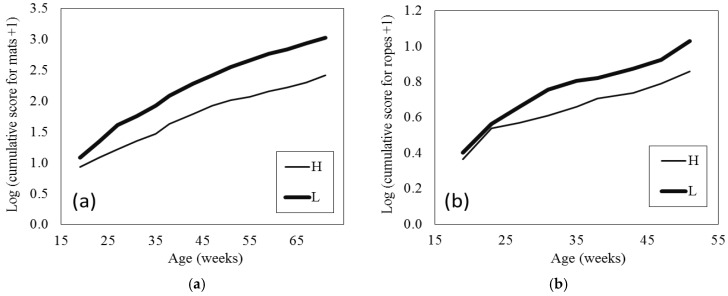
Mean cumulative damage scores (natural log + 1 scale) estimated from linear mixed models (LMMs) for mats (**a**) and ropes (**b**) by breed (H, Hyline Brown; L, Lohmann Brown Classic) and age. Average SED between breeds across ages for (**a**) mat score 0.127 and (**b**) rope score 0.040.

The cumulative damage scores for the pecking mats increased with age (*p* < 0.001), which is to be expected, and L hens accumulated higher scores over time than H hens (age × breed *p* < 0.001; Figure 3a). T hens caused less damage to the mats than NT hens (beak treatment *p* < 0.001 (SED = 0.083); mean ± SE estimated from LMM on log + 1 scale: T 1.78 ± 0.08 score, NT 2.22 ± 0.08 score). To give some context to these scores, based on the back transformed means at the end of the study, mats for NT and L hens were replaced more often than mats for T and H hens, respectively (T 2.4–2.9, NT 3.4–4.3, H 2.1–2.6, and L 3.9–4.9 total mat replacements). The cumulative damage scores for the ropes during age 19 to 51 weeks increased with age (*p* < 0.001), which is to be expected, and L hens caused more damage to the ropes beyond 23 weeks of age than H hens (age × breed *p* = 0.004; Figure 3b). T hens caused less damage to the ropes than the NT (beak treatment *p* < 0.001, (SED = 0.041); mean ± SE estimated from LMM on log + 1 scale: T 0.61 ± 0.03 and NT 0.80 ± 0.03).

### 3.5. Beak Shape

There were no statistically significant effects of breed on the four beak measurements, and for beak tip angle (non-beak treated hens only) there was no statistically significant effect of enrichment. NT hens had longer upper mandible lengths than T hens (*p* < 0.001; mean (mean − SE, mean + SE) estimated from LMM back transformed: NT 2.14 cm (2.12, 2.16), T 1.52 cm (1.51, 1.53)) but there was no statistically significant effect of enrichment. On average, T hens had longer lower mandibles than NT hens (*p* = 0.017; mean (mean − SE, mean + SE) estimated from LMM back transformed: NT 1.31 cm (1.29, 1.32), T 1.36 cm (1.34, 1.37)). For NE hens, there was no difference between T and NT, but T-EE hens had longer lower mandibles than NT-EE hens (beak treatment × enrichment *p* = 0.021 (SED = 0.022); means estimated from LMM back transformed and estimated mean ± SE on transformed scale: T-NE 1.31 cm (0.273 ± 0.018), T-EE 1.40 cm (0.336 ± 0.017), NT-NE 1.31 cm (0.273 ± 0.018), and NT-EE 1.30 cm (0.261 ± 0.018)). The only significant effect for the overhang measure of the beak was due to beak treatment (*p* < 0.001, SED = 0.017), as NT hens had longer overhang lengths for the top mandible than T hens (mean ± SE estimated from LMM: NT 0.258 ± 0.014 cm, T −0.103 ± 0.014 cm, with negative values representing longer lower mandibles).

## 4. Discussion

Overall, H hens performed better than the L hens, regardless of beak treatment. H hens had fewer mortalities, performed fewer bird-to-bird pecks, and had better feather cover. Similarly, the T hens in general fared better than the NT hens. T hens had fewer mortalities (but only due to culling of whole cages due to feather pecking) and better feather cover. In addition, both the H and T hens caused less damage to the extra enrichments than their respective L and NT counterparts. Fewer differences were noted between EE and NE hens. EE hens performed fewer bird-to-bird pecks, but this did not correlate with any overall differences in mortality or feather cover. Furthermore, the extra enrichments had no effect on upper mandible length or our measurement of beak tip sharpness.

Differences in mortality may be due to different management requirements between the breeds. This experiment was carried out on a commercial farm, where the hens in the shed that were not part of the study were Hyline Brown. The staff at this particular farm had more experience with Hyline hens and therefore had probably adopted slight modifications to suit this breed for particular management techniques. Though the diets were formulated to meet age requirements of the hens, they may have originally been formulated to suit the Hyline Brown breed. In addition, management techniques to reduce IP that work for Lohmanns (e.g., reducing light intensity) may not be practical for Hylines as this has had previous detrimental effects for productivity in this particular farm (C Kirk, personal communications). The extent to which the management affected the outcome variables is uncertain, as inherent breed differences have been reported to exist in other research, with significant differences in overall activity levels as well as feather pecking behaviour [36]. Not only do commercially available breeds behave differently, but there is evidence to suggest that feather pecking is a heritable trait [37], as two divergent lines with different propensities for feather pecking have been developed in recent years [9,38]. In addition, certain breeds may be more suited to certain housing environments than other breeds and in our case, the H hens appeared to be better suited to a large furnished cage environment than the L hens, for which the maximum all mortality (8.5%) exceeded the expected rate of 4.9% based on the breed guideline [39]. However, it is important to note that this breed effect was being driven by the L-NT treatment (13.9%), as maximum all mortality for L-T hens (3.1%) was within acceptable breed standards. Although mortality was very low in the H group, the H-NT hens (2.0%) appeared to have more mortality than H-T hens (1.0%), though this was marginally significant. Though a mortality threshold (*i.e.*, humane endpoint) was implemented for individual cages during this experiment, this would probably not be the case on a commercial farm. Admittedly, it is uncertain how many more hens would have died in the two affected cages had there not been an intervention. Commercial producers may opt for other interventions (e.g., re-beak trim, reducing light intensity, separating mildly affected hens, *etc.*) or may not do anything at all. Regardless, any of those interventions may not reduce IP and may prolong the suffering of affected cages.

Though a relatively considerable amount of feather damage and mortality due to cannibalism was observed in this experiment, the overall level of observed IP does not appear to correspond to these measures. However, there is some evidence to suggest that only a small proportion of hens (*i.e.*, <12%) in any given group actually perform the majority of IP behaviour [40,41], and thus this may be difficult to observe in general. In this type of commercial system, video surveillance to collect behaviour data remotely is not easily accomplished. Therefore, direct behaviour observations were the best option, though this type of data collection presented its own problems as well (*i.e.*, limited time available to observe and disruption to normal behaviour). Hens did not perform much pecking behaviour during observations, though differences were still detectable when all types of bird-to-bird pecks from focal observations were combined into one analysis. However, these hens had not been previously habituated to human presence, so the presence of the observer may have influenced behaviour. Though gentle feather pecking may not be directly harmful to the recipient hen’s welfare, its presence in a repetitive manner may represent a welfare problem in itself, as it is generally observed occurring in a stereotyped manner [42,43]. In addition, some research has suggested that gentle feather pecking is correlated to severe or vigorous feather pecking [12], however others have found no such link [43]. In addition, one study [44] demonstrated a difference in morphology between gentle and severe feather pecks, with severe feather pecks being most similar to foraging pecks. L hens were observed to perform more bird-to-bird pecks, which may have played a role in the differences observed for mortality and feather cover. They also appeared to be more active, both in general, as reflected in the rate of bird changes during focal observations, as well as in regards to bird-to-bird pecking. Similarly, [45] found that hens from a high feather pecking line were more active than those from a low feather pecking line, suggesting a link between hyperactivity and the genetic basis for feather pecking.

Not surprisingly, the NT hens caused more feather damage, had more deaths (maximum total and IP mortalities), and caused more damage to the extra enrichments. It is well described in the literature that beak treatment can reduce mortality and feather damage [25]. NT hens were most likely more efficient with their beaks and so are likely to cause more damage with the same number of pecks as T hens. Another study [46] reported reduced feeding efficiency in beak treated hens, as measured by percent of pecks that resulted in successful acquisition of feed pellets, for beak treated hens compared to hens with intact beaks. A separate study reported a positive correlation between ground pecks and feather pecks within large groups of hens [40]. Our data also suggest that hens that cause more damage to the extra enrichments (in theory, either by pecking more frequently or more severely) also appear to perform more bird-to-bird pecking. Ideally, pecking at enrichments would reduce the time spent feather pecking based on a shift in time budgets or based on a reduction in foraging frustration, and improve feather cover. When presented with enrichments, hens from other studies were observed to perform less feather pecking behaviour [47] and aggression [18,19]. However, this did not appear to be the case in this current experiment, given the lack of an overall enrichment effect on feather cover, despite evidence to suggest that hens were in fact using the extra enrichments to some degree. Though there was a significant effect of extra enrichment presence for NT hens, it did not have the expected effect. In fact, NT-EE hens appeared to have worse feather cover than NT-NE hens, though this effect was marginally significant and may not reflect true biological differences. In this experiment, the ends of the ropes had been cauterised, which differed from other studies that used similar ropes [22,48]. This may have impacted on their overall use and subsequent effect on the outcomes we measured.

Though the hens were using the ropes and mats (evident during behavioural observations as well as indicated by the damage to the extra enrichments), it did not appear that the blunting boards attracted much attention. Therefore, it is unlikely that the board would have had the chance to affect beak morphology, and this was reflected in the results. Generally, this type of device would be better suited on the bottom of the feed trough, where the hens would have to come in contact with the abrasive surface on a more regular basis [32,33]. It was not feasible to permanently alter the cages in any way as this experiment was carried out on a commercial farm. In addition, from a practical point of view, placing this type of enrichment on the bottom of the feed trough would not easily suit a trough with a chain feeder, limiting its potential application to barns with hopper feeders. A surprising result was the longer bottom mandibles observed in the T-EE hens compared to the NT-EE hens (without the same difference noted in the NE hens). This result was still evident even after removing some outlying data points, yet we are not certain as to why this result would exist. However, it is possible that this result was a consequence of pre-existing differences between treatments.

## 5. Conclusions

In conclusion, we were able to show that breed choice plays an important role in regards to successful housing of laying hens. H hens had fewer mortalities overall and the increase in IP-related mortality for NT hens was not as evident for H hens as it was for L hens. The levels of IP related mortality and feather damage observed with the L hens were not within acceptable levels according to the farm staff (C Kirk, personal communication) as well as the standards set out in the breed guidelines [39], and represent a high degree of insult to animal welfare. However, these results appear to be mainly driven by the L-NT treatment combination, though L-T mortality was still higher than H-T mortality. Not surprisingly, beak treatment was effective at reducing (maximum) mortality and improving feather cover, which are important welfare parameters for commercial production. Though the mat and rope enrichments were used by the hens and reduced bird-to-bird pecking, overall they did not affect any of the main welfare outcomes (*i.e.*, mortality and feather cover). Presumably, an increase in behavioural repertoire would inherently improve welfare, but some modifications would be required to improve the extra enrichments’ benefits prior to commercial applicability.

## Figures and Tables

**Table 1:** Ethogram for behavioural observations during scan and focal sampling. All incidences of any birds observed performing each of the oral behaviours listed below were recorded.

Behaviour	Description
Gentle feather peck **^I,B^**	Furtive, deliberate, often repeated pecks at another bird’s feather where the recipient usually does not react.
Vigorous feather peck **^I,B^**	Similar to gentle feather pecks, however, includes grasping and pulling of the feather and may include feather removal. Recipient often reacts, squawks or withdraws.
Cannibalistic peck **^I,B^**	Pecks at (or causing) bloody wounds, tissue damage or removal. Directed towards ANY part of the body EXCEPT for the vent (see vent peck) or toes (see toe peck).
Vent peck **^I,B^**	Pecks to the vent area.
Toe peck **^B^**	Pecks at another bird’s toes.
Aggressive peck **^B^**	Overt, rapid, forceful, usually downward directed pecks, usually directed toward recipient’s head or back of neck. Usually not overly repetitive, recipient reacts immediately.
Rope peck **^F,E^**	Any peck directed at any of the ropes.
Board peck **^F,E^**	Any peck directed at the blunting board.
Mat peck **^F,E^**	Any peck directed at the pecking mat.
Other peck **^F^**	Any other peck not listed above, including feeding, drinking, beak pecking, self-pecks, pecks directed to cage fixtures.
Receive peck **^F^**	Any type of peck that the focal bird receives.

**^I^** Behaviours classed as a part of injurious pecking (IP); **^B^** Bird-to-bird behaviours analysed together as a group (focal sampling only); **^F^** Behaviours recorded during focal sampling only, and performed by the focal hen; **^E^** Behaviours directed at extra enrichments analysed as a group (focal sampling only).

**Table 2:** Raw mean percentage (%) total mortality (16–71 weeks) and estimated means ± SE on the logit scale together with *p* values (SED) for statistically significant fixed effects from the generalised mixed models (GLMMs). Maximum data includes all hens from the two cages that were removed from the study due to surpassing the pecking related threshold, whilst minimum data only includes hens from these cages that died before these cages were removed.

Effect	Minimum All Deaths	*p*-Value (SED)	Maximum All Deaths	*p*-Value (SED)	Minimum IP-Related Deaths	*p*-Value (SED)	Maximum IP-Related Deaths	*p*-Value (SED)
Breed								
H	1.52% (−4.26 ± 0.20)	0.002 (0.21)	1.52% (−4.80 ± 0.44)	<0.001 (0.20)	0.12% (−7.03 ± 0.71)	0.003 (0.69)	0.12% (−8.88 ± 1.07)	<0.001 (0.68)
L	2.97% (−3.54 ± 0.16)		8.52% (−3.13 ± 0.41)		0.86% (−4.95 ± 0.36)		6.41% (−5.31 ± 0.87)	
Beak								
T	2.07% (−4.06 ± 0.20)	0.569 (0.21)	2.07% (−4.60 ± 0.44)	<0.001 (0.20)	0.35% (−6.34 ± 0.63)	0.210 (0.67)	0.35% (−8.19 ± 1.02)	<0.001 (0.66)
NT	2.42% (−3.74 ± 0.l7)		7.97% (−3.33 ± 0.42)		0.63% (−5.64 ± 0.48)		6.17% (−6.00 ± 0.92)	
Breed × Beak								
H-T	1.02% (−4.61 ± 0.30)	0.039 (0.29)	1.02% (−5.15 ± 0.49)	0.013 (0.28)	0.08% (−7.41 ± 1.09)	0.942 (0.92)	0.08% (−9.29 ± 1.36)	0.025 (0.90)
H-NT	2.03% (−3.91 ± 0.23)		2.03% (−4.45 ± 0.45)		0.16% (−6.64 ± 0.78)		0.16% (−8.47 ± 1.12)	
L-T	3.13% (−3.52 ± 0.20)		3.13% (−4.04 ± 0.44)		0.63% (−5.26 ± 0.46)		0.63% (−7.09 ± 0.93)	
L-NT	2.81% (−3.57 ± 0.20)		13.91% (−2.21 ± 0.41)		1.09% (−4.65 ± 0.38)		12.19% (−3.52 ± 0.85)	
Beak × Enr.								
T-NE	2.66% (−3.84 ± 0.24)	0.144 (0.30)	2.66% (−4.36 ± 0.46)	0.037 (0.28)	0.47% (−5.92 ± 0.65)	0.609 (0.85)	0.47% (−7.74 ± 1.03)	0.375 (0.82)
T-EE	1.48% (−4.29 ± 0.27)		1.48% (−4.83 ± 0.48)		0.23% (−6.75 ± 0.87)		0.23% (−8.64 ± 1.19)	
NT-NE	2.34% (−3.79 ± 0.22)		7.81% (−3.41 ± 0.43)		0.70% (−5.45 ± 0.54)		6.17% (−5.88 ± 0.95)	
NT-EE	2.50% (−3.69 ± 0.21)		8.13% (−3.25 ± 0.43)		0.55% (−5.83 ± 0.64)		6.17% (−6.12 ± 1.00)	

**Table 3:** Percentages (%) of each type of peck out of the total number of observed pecks during focal behaviour sampling.

Peck Type	Percentage of Observed Pecks (%)
Gentle feather peck	5.29%
Vigorous feather peck	0.94%
Cannibalistic peck	0%
Vent peck	0%
Toe peck	0.15%
Aggressive peck	0.48%
Rope peck	1.73%
Board peck	0.31%
Mat peck	0.97%
Other peck	84.27%
Receive peck	5.86%

**Table 4:** Estimated mean (mean − SE, mean + SE) back transformed on to rate (pecks or changes per minute) scale together with *p* values for statistically significant fixed main effects from the linear mixed models (LMMs).

Effect	Bird-to-Bird Pecks	*p*-Value	Bird Changes	*p*-Value
Breed				
H	0.042 (0.016, 0.073)	0.015	1.291 (1.221, 1.364)	<0.001
L	0.163 (0.115, 0.223)		1.919 (1.830, 2.012)	
Beak Treatment				
T	0.124 (0.083, 0.173)	0.253	1.460 (1.385, 1.538)	0.048
NT	0.067 (0.036, 0.105)		1.719 (1.635, 1.806)	
Enrichment				
NE	0.153 (0.106, 0.209)	0.033	1.562 (1.483, 1.643)	0.694
EE	0.048 (0.021, 0.081)		1.612 (1.531, 1.695)	
Start Location				
Nest	0.030 (0.002, 0.065)	<0.001	1.553 (1.455, 1.654)	0.046
Board	0.105 (0.061, 0.160)		1.814 (1.707, 1.926)	
Mat	0.022 (−0.004, 0.054)		1.586 (1.488, 1.688)	
Rope	0.330 (0.239, 0.447)		1.408 (1.316, 1.504)	

## Abbreviations

The following abbreviations are used in this manuscript:

IP: injurious pecking
H: Hyline Brown
L: Lohmann Brown Classic
T: beak treated
NT: non-beak treated
NE: no extra enrichments
EE: extra enrichments
